# Modification of Threonine-1050 of SlBRI1 regulates BR Signalling and increases fruit yield of tomato

**DOI:** 10.1186/s12870-019-1869-9

**Published:** 2019-06-13

**Authors:** Shufen Wang, Jianwei Liu, Tong Zhao, Chenxi Du, Shuming Nie, Yanyu Zhang, Siqi Lv, Shuhua Huang, Xiaofeng Wang

**Affiliations:** State Key Laboratory of Crop Stress Biology for Arid Areas, College of Horticulture, Northwest A&F University, Yangling, 712100 Shaanxi China

**Keywords:** Tomato, SlBRI1, Phosphorylation site, Agronomic trait, BR signal

## Abstract

**Background:**

Appropriate brassinosteroid (BR) signal strength caused by exogenous application or endogenous regulation of BR-related genes can increase crop yield. However, precise control of BR signals is difficult and can cause unstable effects and failure to reach full potential. Phosphorylated BRASSINOSTEROID INSENSITIVE1 (BRI1), the rate-limiting receptor in BR signalling, transduces BR signals, and we recently demonstrated that modifying BRI1 phosphorylation sites alters BR signal strength and botanical characteristics in Arabidopsis. However, the functions of such phosphorylation sites in agronomic characteristics of crops remain unclear.

**Results:**

In this work, we investigated the roles of tomato SlBRI1 threonine-1050 (Thr-1050). *SlBRI1* mutant *cu3*^*-abs1*^ plants expressing SlBRI1 with a non-phosphorylatable Thr-1050 (T1050A), with a wild-type SlBRI1 transformant used as a control, were examined. The results showed enhanced autophosphorylation of SlBRI1 and BR signal strength for *cu3*^*-abs1*^ harbouring T1050A, which promoted yield through increased plant expansion, leaf area, fruit weight and fruit number per cluster but reduced nutrient contents, including ascorbic acid and soluble sugar levels. Moreover, plant height, stem diameter, and internodal distance were similar between the transgenic plants.

**Conclusion:**

Our results reveal the biological role of Thr-1050 in tomato and provide a molecular basis for establishing high-yield crops by precisely controlling BR signal strength via phosphorylation site modification.

**Electronic supplementary material:**

The online version of this article (10.1186/s12870-019-1869-9) contains supplementary material, which is available to authorized users.

## Background

Brassinosteroids (BRs), endogenous plant hormones with physiological activity at nanomolar concentrations, promote plant growth through involvement in processes such as germination, leaf morphogenesis, plant architecture, flowering, male fertility and senescence [1–3]. As a specific BR receptor, BRASSINOSTEROID INSENSITIVE1 (BRI1) functions indispensably in BR signal transduction in plants [4]. BRI1 is a leucine-rich repeat receptor kinase comprising an extracellular domain, transmembrane domain, and cytoplasmic domain [5]; the cytoplasmic domain contains the juxtamembrane region (JM), the serine/threonine/tyrosine kinase domain (KD), and the C-terminal (CT) domain [6]. In the BR signal transduction pathway, BRs bind to the extracellular domain of BRI1, promoting heterodimerization between BRI1 and its coreceptor BRI1-ASSOCIATED RECEPTOR KINASE1 (BAK1), which in turn activates sequential transphosphorylation of the KD domain of both proteins [7]. This process ultimately results in activation of BRASSINAZOLERESISTANT1 (BZR1) and BRI1-EMS SUPPRESSOR1 (BES1) [8–10], both of which function as transcription factors to regulate the expression of BR-responsive genes [11–13].

Biochemical and genetic studies in Arabidopsis have demonstrated that BRI1 plays a critical role in plant growth and development. In Arabidopsis, most of mutant *bri1* alleles are BR insensitive and display a dwarf phenotype consisting of curled leaves, delayed growth, and male sterility [14]. Overexpression of *BRI1* causes increased petiole length and sensitivity to BRs [15]. In addition, site-directed mutagenesis of BRI1 phosphorylation sites differentially affects both plant growth and BR signalling. Most of the phosphorylation sites of BRI1 are located in the KD domain, such as Tyr-956, Thr-1039, Thr-1049, Ser-1044, and Thr-1045, and exhibit strong functions in BR signalling and plant growth. Indeed, preventing phosphorylation of these residues attenuates BR signalling and disturbs plant growth. However, unphosphorylated Ser-1168 and Ser-1172 mutants (CT domain) exhibit only slightly inhibited leaf growth but greatly reduced seed yields. In the JM domain, Tyr-831 is involved in the regulation of leaf growth and flowering time. Ser-891 is associated with deactivation mechanisms, as transformants harbouring unphosphorylated Ser-891 present enhanced BR signalling and are larger than those harbouring wild-type BRI1 [16–18]. Notably, a mutant with non-phosphorylatable Ser-1042 shows dramatically decreased kinase activity and a semi-dwarf phenotype but has normal seed yields, resulting in enormous potential for increasing crop production. All these findings substantiate a novel approach for regulating economic yields precisely via modification of specific phosphorylation sites of BRI1 in crop species.

Accordingly, numerous biological functional analyses of BRI1 orthologues among different plant species have been performed. In rice, *OsBRI1* (*d61*) loss-of-function mutants display relatively shorter plant height and erect leaves, with had little effect on fertility; because of the greater photosynthetic capacity and leaf area index of *OsBRI1* mutants under dense planting conditions, *OsBRI1* is a promising factor for increasing yields [19]. *ZmBRI1*-RNAi transgenic maize plants also display a dwarf stature, shortened internodes, and twisted leaves, and BR signalling is compromised [20]. The barley mutant *uzu*, which is caused by suppression of *HvBRI*, presents a semi-dwarf stature and pathogen resistance, which is beneficial for high yields [21–23]. Additionally, heterologous expression of *TaBRI1* in Arabidopsis may promote plant germination, flowering and seed yield [24]. In strawberry, the level of *FaBRI1* mRNA expression was found to increase rapidly during the ripening stage, whereas suppressing *FaBRI1* in de-greening fruit significantly slowed the development of red colouring [25]. Overall, these studies highlight the valuable potential of *BRI1* in agricultural production, and controlling BR signal strength via *BRI1* may be an effective approach to increase yield.

Tomato is a major horticultural crop and a model plant among berry-producing plants in plant molecular biology research. Tomato *SlBRI1* was first characterized in the *curl-3* mutant, which showed a phenotype similar to that of the Arabidopsis *bri1* mutant. Overexpression of *SlBRI1* in tomato enhanced the endogenous BR signal intensity and improved major agricultural traits such as fruit yield and quality [26]. Protein sequence alignments have revealed that the KD domains as well as the phosphorylation sites are highly conserved between SlBRI1 and BRI1 [27], though heterologous expression of *SlBRI1* in Arabidopsis *bri1* mutants did not fully rescue the mutation [14, 28]. These results suggest that different regulatory mechanisms may exist between *SlBRI1* and *BRI1*. The *cu3*^*-abs1*^ mutant is a weak *SlBRI1* mutant with a missense mutation in the kinase domain. Moreover, site-directed mutagenesis of SlBRI1 Thr-1054 in the *cu3*^*-abs1*^ background failed to rescue the dwarf mutation and caused an even more severe dwarf phenotype, revealing the critical role of this phosphorylation site in BR signal transduction and biological characteristics in tomato [29]. However, functional analyses of SlBRI1 phosphorylation sites have been restricted to only Thr-1054, without considering important agronomic values, and the roles of other phosphorylation sites in BR signalling and agronomic characteristics remain unclear.

Accordingly, we investigated the biological function of threonine-1050 (Thr-1050) in the KD domain of tomato SlBRI1, which is equivalent to BRI1 Thr-1045. We generated a T1050A mutant of SlBRI1 via threonine-1050 to alanine substitution, developed transgenic *cu3*^*-abs1*^ plants expressing either T1050A or wild-type SlBRI1, and compared the degree of mutant phenotype restoration. Our results show that compared with SlBRI1, expression of T1050A resulted in stronger BR signal strength, higher autophosphorylation levels, more vigorous vegetative development, higher yields, and lower fruit nutrient content. These results demonstrate a unique negative regulatory function of Thr-1050 phosphorylation in tomato growth and development, which will be valuable for revealing the mechanisms involved in tomato BR signalling and for improving crop performance via precise modification of specific phosphorylation sites.

## Results

### SlBRI1 Thr-1050-Ala promotes plant vegetative growth

**Fig. 1:**
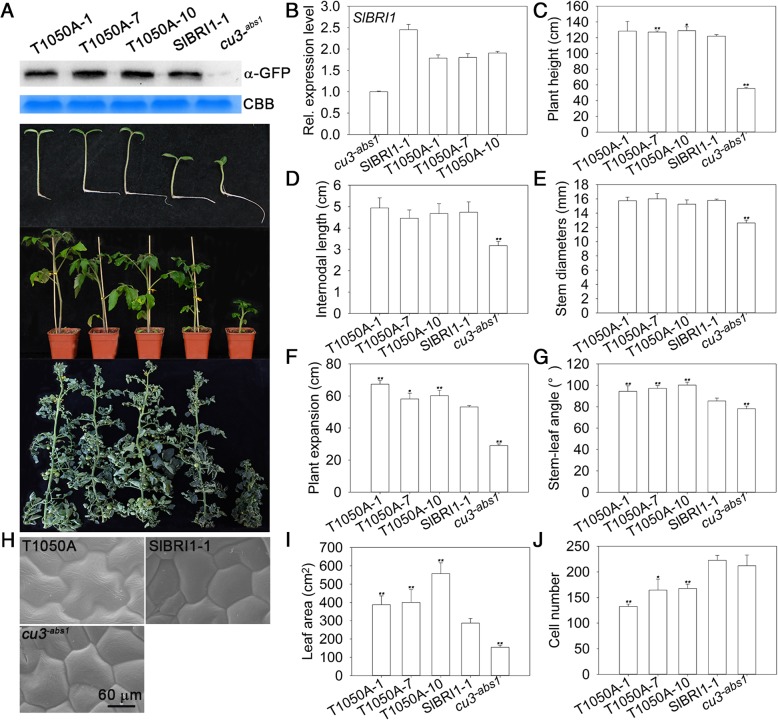
Dephosphorylation of Thr-1050 improves plant vegetative growth. **a** Top, western blot analysis of transgenic protein expression using anti-green fluorescent protein (GFP) antibodies. CBB, Coomassie brilliant blue. Bottom, phenotypes of plants at the germination stage (8 days after sowing), seedling stage (40 days after sowing), and maturation stage (120 days after sowing). **b** Relative transcript levels of *SlBRI1* by qRT-PCR. **c** Plant height of plants at the maturation stage. **d** Internodal length and (**e**) stem diameters of the second flower node. **f** Plant expansion at the maturation stage. **g** Stem-leaf angle, (**h**) ultrastructure by scanning electron microscopic under a 1500x-magnified visual field, and (**i**) leaf area of the sixth leaf. **j** Cell number per unit of the sixth leaf under a 300x-magnified visual field. Data are the means ± SDs of at least 5 independent biological samples. *Asterisks* indicate significant differences compared with P_*SlBRI1*_::SlBRI1-GFP plants (*P < 0.05; **P < 0.01; Student’s t-test)

To determine how Thr-1050 phosphorylation extensively affects plant growth and development, transgenic tomato plants expressing SlBRI1 or T1050A under the control of the native promoter were generated. Transgenic line P_*SlBRI1*_::SlBRI1-green fluorescent protein (GFP)-1 (SlBRI1–1 hereafter), which showed a similar SlBRI1 expression level and phenotype with other P_*SlBRI1*_::SlBRI1-GFP lines, as well as *cu3*^*-abs1*^, was selected as the control (Additional file 1: Figure S1). The phenotype of P_*SlBRI1*_::T1050A-GFP was evaluated and compared with that of *cu3*^*-abs1*^ and SlBRI1–1. To reduce experimental error and determine the physiological significance of Thr-1050, transgenic lines P_*SlBRI1*_::T1050A-GFP-1, P_*SlBRI1*_::T1050A-GFP-7, P_*SlBRI1*_::T1050A-GFP-10 (T1050A-1, T1050A-7, and T1050A-10), which presented SlBRI1 expression levels similar to those of SlBRI1–1, were selected; the transcript levels of *SlBRI1* in these transgenic lines were more than 2-fold greater than those in *cu3*^*-abs1*^ (Fig. 1a, b).

During the vegetative growth stage, the growth trend of P_*SlBRI1*_::T1050A-GFP was better than that of P_*SlBRI1*_::SlBRI1-GFP and *cu3*^*-abs1*^ (Fig. 1a). Plant height, internodal length, and stem diameter were similar between P_*SlBRI1*_::T1050A-GFP and P_*SlBRI1*_::SlBRI1-GFP; however, the values were 2.3-, 1.5-, and 1.2-fold greater than those of *cu3*^*-abs1*^, respectively (Fig. 1c, d, e). Overall, P_*SlBRI1*_::T1050A-GFP exhibited the greatest degree of plant expansion and largest stem-leaf angle: plant expansion was 1.2- and 2.1-fold that of P_*SlBRI1*_::SlBRI1-GFP and *cu3*^*-abs1*^, respectively, and the stem-leaf angle was 1.1- and 1.2-fold that of P_*SlBRI1*_::SlBRI1-GFP and *cu3*^*-abs1*^, respectively (Fig. 1f, g). To compare differences in leaf development, the leaf area and CO_2_ assimilation rates were analysed. The leaf area of P_*SlBRI1*_::T1050A-GFP was 1.6-fold greater than that of P_*SlBRI1*_::SlBRI1-GFP; this difference was mostly due to the larger cell size, as the cell number of P_*SlBRI1*_::T1050A-GFP was 70% that of P_*SlBRI1*_::SlBRI1-GFP (Fig. 1h, i, j). However, CO_2_ assimilation rates of all plants were the same (Additional file 2: Figure S2). Taken together, these results indicate that dephosphorylation of Thr-1050 can rescue the functions of SlBRI1 in terms of plant height and photosynthesis and even promote plant expansion and leaf cell expansion during the vegetative growth stage.

### Dephosphorylation of Thr-1050 improves tomato yields

**Fig. 2:**
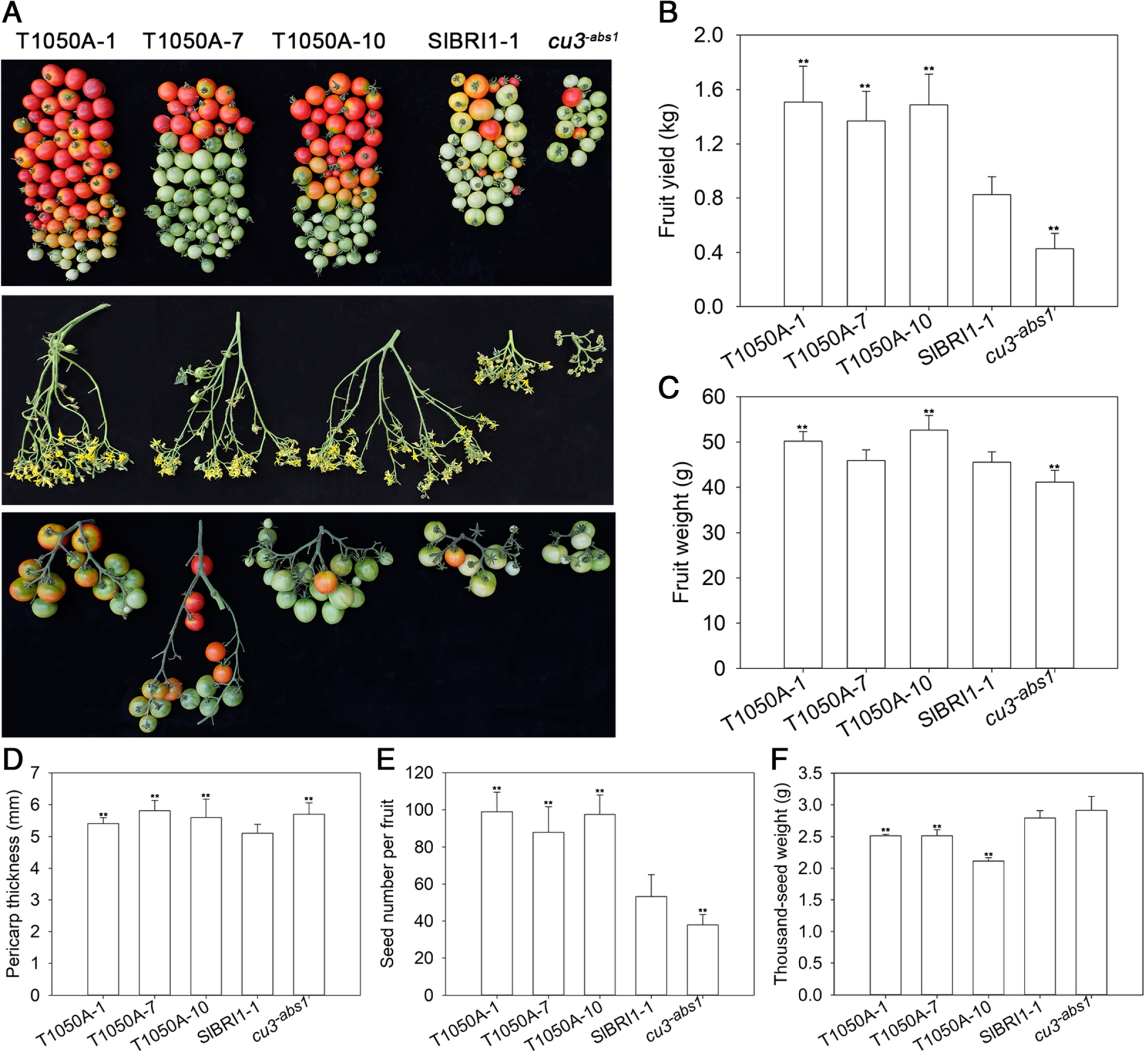
Dephosphorylation of Thr-1050 promotes tomato yield. **a** Top, fruits from the first to the fourth fruit node. Middle, phenotypes of the third inflorescence. Bottom, phenotypes of the third cluster. **b** Fruit yield per plant. **c** Individual fruit weight at the red ripening (RR) stage. **d **Pericarp thickness of the fruit at the yellow ripening stage. **e** Seed number per fruit. **f** Thousand-seed weight. Data are the means ± SDs of 15 independent biological samples. *Asterisks* indicate significant differences compared with P_*SlBRI1*_::SlBRI1-GFP plants (*P < 0.05; **P < 0.01; Student’s t-test)

To assess the effects of Thr-1050 on fruit yield, we investigated correlating factors, including flowering time, fruit weight, fruit number per cluster, pericarp thickness, seed number, and thousand-seed weight. For example, plants expressing T1050A flowered 1 week earlier than did P_*SlBRI1*_::SlBRI1 plants and 2 weeks earlier than did *cu3*^*-abs1*^ plants. The yield per plant for P_*SlBRI1*_::T1050A-GFP was 1.8- and 3.4-fold that for P_*SlBRI1*_::SlBRI1-GFP and *cu3*^*-abs1*^, respectively, and these differences were due mainly to the different fruit number per cluster (Fig. 2 a, b). In addition, the fruit weight for P_*SlBRI1*_::T1050A-GFP was 9% heavier than that for P_*SlBRI1*_::SlBRI1-GFP because the pericarp of the former was 10% thicker (Fig. 2 c, d). Regarding seeds per fruit, those expressing T1050A contained 1.8-fold more than those of P_*SlBRI1*_::SlBRI1-GFP; however, the thousand-seed weight for P_*SlBRI1*_::T1050A-GFP was only slightly lower than that for P_*SlBRI1*_::SlBRI1-GFP (Fig. 2 e, f). Thus, Thr-1050 appears to play a role in tomato yield.

### SlBRI1 Thr-1050-Ala alters fruit quality

**Fig. 3:**
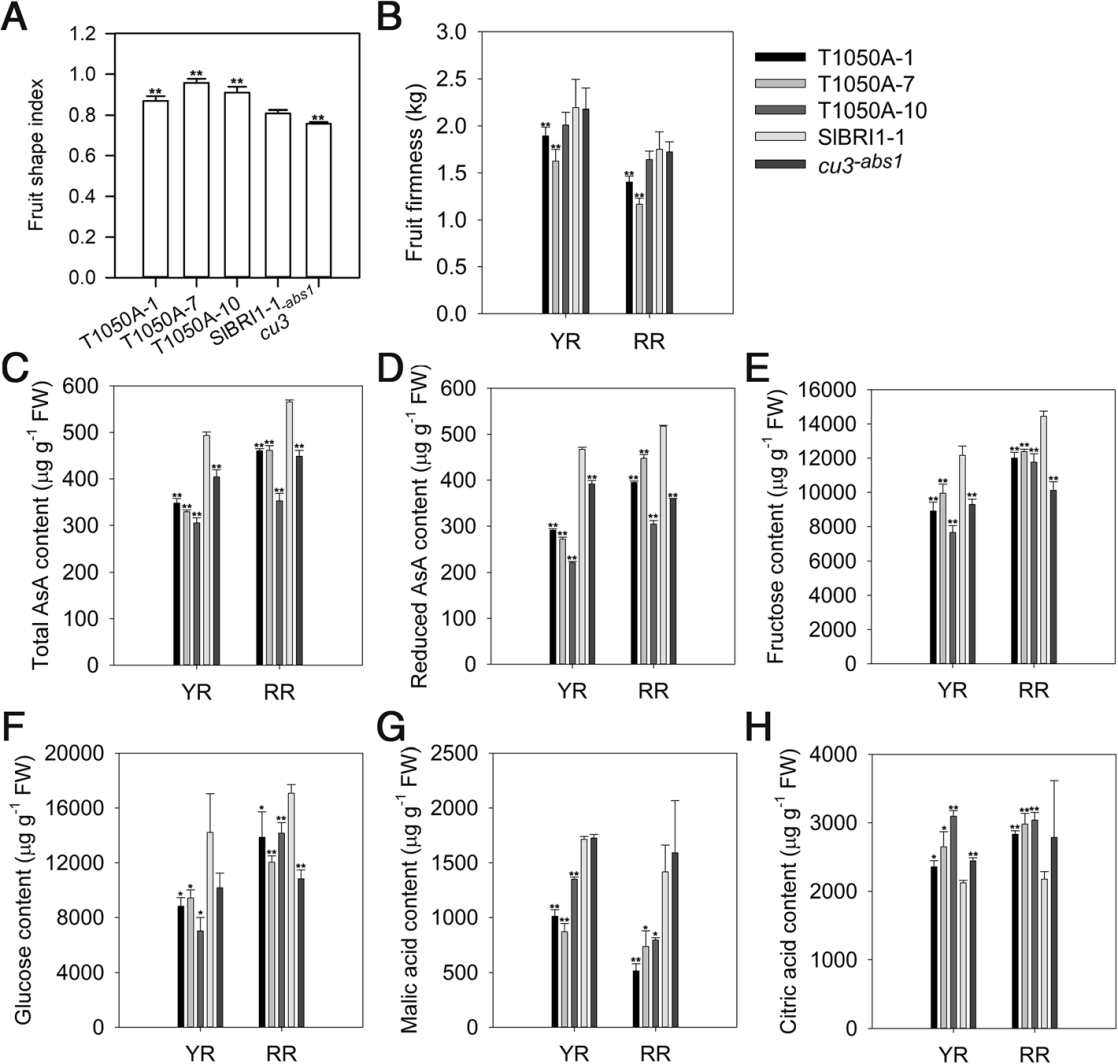
Dephosphorylation of Thr-1050 alters fruit quality. **a** Fruit shape index at the breaker stage. **b** Fruit firmness at yellow ripening (YR) and red ripening (RR) stages. **c** and (**d**) Contents of total ascorbic acid (AsA) (**c**) and reduced AsA (**d**). **e**, (**f**), (**g**) and (**h**) Contents of fructose (**e**), glucose (**f**), malic acid (**g**), and citric acid (**h**) measured using HPLC. Date for (**a**) and (**b**) are the means ± SDs of 15 independent biological samples; data for (**c**) to (**h**) are the means ± SDs of 3 independent biological samples. FW = fresh weight. *Asterisks* indicate significant differences compared with P_*SlBRI1*_::SlBRI1-GFP plants (*P < 0.05; **P < 0.01; Student’s t-test)

We also investigated the role of Thr-1050 in fruit quality. Both SlBRI1 and T1050A were able to rescue the fruit quality of *cu3*^*-abs1*^. Compared with P_*SlBRI1*_::SlBRI1-GFP, the fruits of P_*SlBRI1*_::T1050A-GFP presented a greater shape index but were less firm during the ripening stage (Fig. 3 a, b). The total ascorbic acid (AsA) content in fruits were 66% of those of P_*SlBRI1*_::SlBRI1-GFP fruits at the yellow ripening (YR) stage and 75% of those in the same fruits at the red ripening (RR) stage. AsA contents were 56 and 74% lower than those of the fruits of P_*SlBRI1*_::SlBRI1-GFP at the YR and RR stages, respectively (Fig. 3 c, d). Additionally, contents of soluble sugars such as fructose and glucose were measured. The fructose content in the fruits of P_*SlBRI1*_::T1050A-GFP were decreased by 27 and 17% at the YR and RR stages, respectively, and the glucose content also decreased by 41 and 22%, respectively (Fig. 3 e, f). Furthermore, the contents of organic acids such as malic acid and citric acid were analysed. The results showed that the malic acid contents in the fruits of P_*SlBRI1*_::T1050A-GFP were 37 and 48% lower than those in the fruits of P_*SlBRI1*_::SlBRI1-GFP at the YR and RR stages, respectively; however, citric acid contents were 27 and 36% higher (Fig. 3 g, h). No significant differences in soluble solid contents among the transgenic lines were observed (Additional file 3: Figure S3).

### Phosphorylation of SlBRI1 Thr-1050 affects BR Signalling

**Fig. 4:**
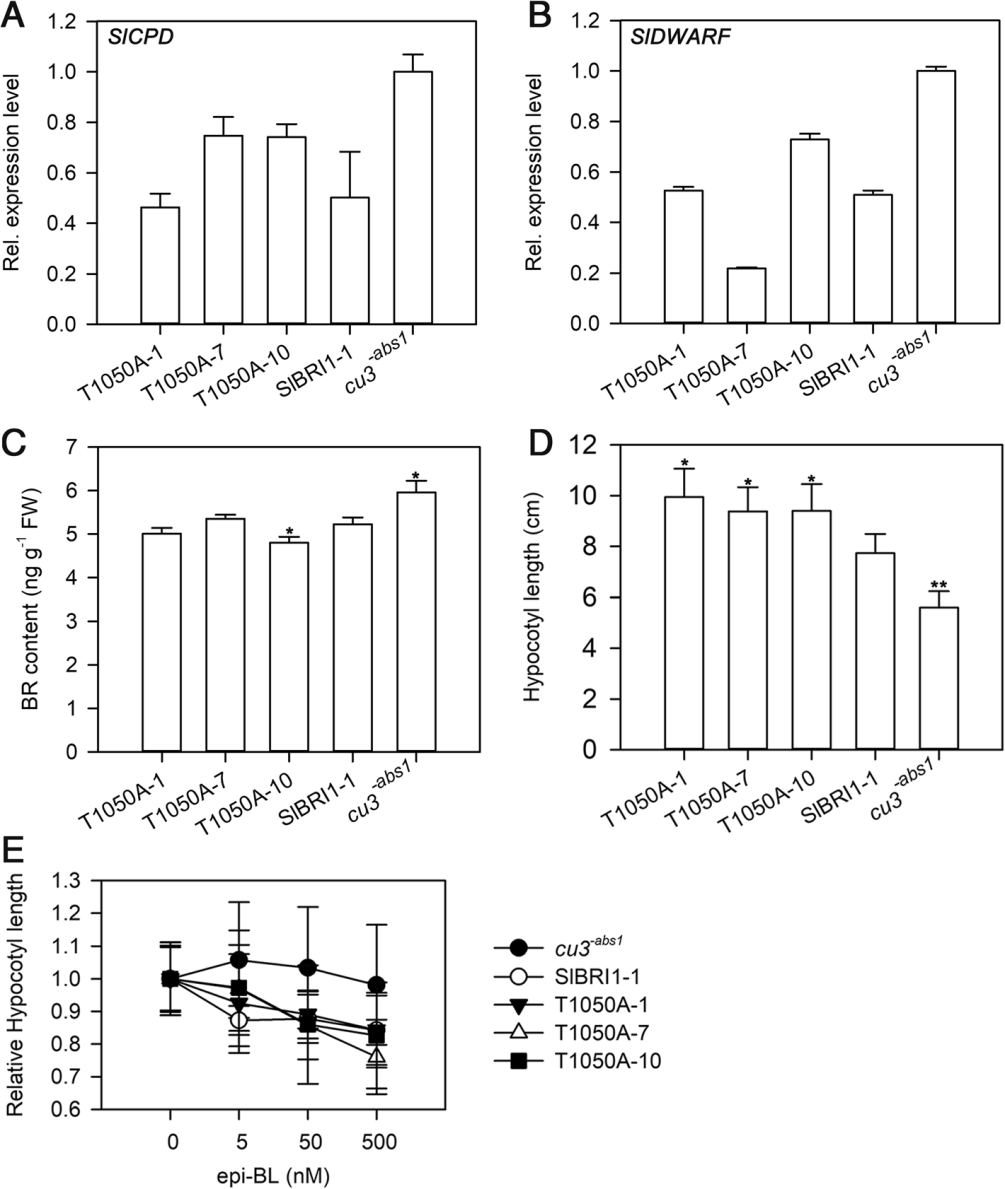
Dephosphorylation of Thr-1050 affects BR signalling. **a** and (**b**) Relative transcript levels of BR signalling marker genes *SlCPD* and *SlDWARF* were tested by qRT-PCR. **c** BR content in the third leaf measured using a BR ELISA Kit. Data are the means ± SDs of 3 independent biological samples. **d** Hypocotyl length of seedlings grown in the dark for 9 days on the surface of the medium. **e** Dose-response curves of relative hypocotyl length of seedlings grown in the dark for 9 days on the surface of media supplemented with increasing concentrations of epibrassinolide (epi-BL). Data for (**d**) and (**e**) are the means ± SDs of 15 independent biological samples. *Asterisks* indicate significant differences compared with P_*SlBRI1*_::SlBRI1-GFP plants (*P < 0.05; **P < 0.01; Student’s t-test)

As a BR receptor, SlBRI1 plays a key role in BR signalling. To determine whether Thr-1050 phosphorylation regulates tomato development by affecting BR signalling, BR biosynthesis, hypocotyl elongation in the dark, and BR sensitivity of seedlings were evaluated to compare BR signal intensities among *cu3*^*-abs1*^, P_*SlBRI1*_::SlBRI1-GFP, and P_*SlBRI1*_::T1050A-GFP. The expression levels of tomato BR biosynthesis genes *SlCPD* and *SlDWARF* were assessed by quantitative real-time PCR (qRT-PCR). Although the expression levels of *SlCPD* and *SlDWARF* were higher in *cu3*^*-abs1*^ than in the transgenic lines, there were no clear differences between P_*SlBRI1*_::SlBRI1-GFP and P_*SlBRI1*_::T1050A-GFP (Fig. 4 a, b). Figure 4 c showed that BR contents in the transgenic lines were similar but were approximately 18% lower than those of *cu3*^*-abs1*^. In addition, BR sensitivity of tomato plants was examined under four increasing concentrations of exogenous 24-epibrassinolide (epi-BL). As shown in Fig. 4 e, *cu3*^*-abs1*^ was insensitive to epi-BL; in contrast, hypocotyl length in P_*SlBRI1*_::SlBRI1-GFP and P_*SlBRI1*_::T1050A-GFP was similarly reduced by epi-BL. When treated with 500 nM epi-BL, hypocotyl length in P_*SlBRI1*_::SlBRI1-GFP and P_*SlBRI1*_::T1050A-GFP decreased by 16 and 19%, respectively. Thus, analogous to SlBRI1, Thr-1050 was able to rescue BR signalling in plants. However, the result of hypocotyl elongation in the dark showed that P_*SlBRI1*_::T1050A-GFP exhibited the longest hypocotyl length, which was approximately 71 and 24% longer than that of *cu3*^*-abs1*^ and P_*SlBRI1*_::SlBRI1-GFP (Fig. 4 d). This result demonstrate that BR signalling in P_*SlBRI1*_::T1050A-GFP was stronger than that in P_*SlBRI1*_::SlBRI1-GFP.

### Phosphorylation of SlBRI1 Thr-1050 influences autophosphorylation of SlBRI1

**Fig. 5:**
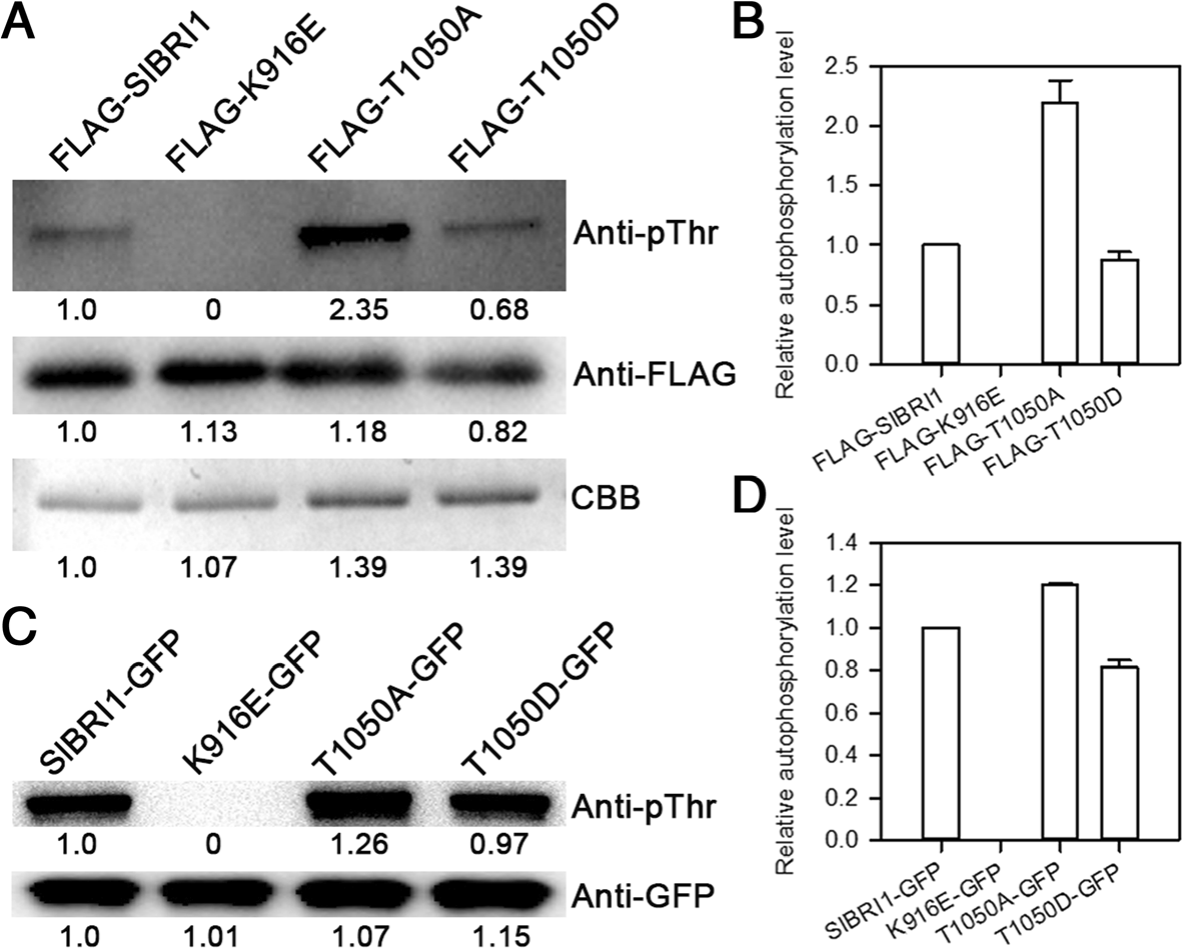
Dephosphorylation of Thr-1050 influences SlBRI1 autophosphorylation. **a** Autophosphorylation level of SlBRI1 in vitro. Autophosphorylation activity of recombinant FLAG-SlBRI1, FLAG-T1050A, FLAG-T1050D, and FLAG-K916E proteins was detected using anti-pThr antibodies, and aliquots of the recombinant proteins were detected using anti-FLAG antibodies and western blotting. Coomassie brilliant blue (CBB) staining shows loading. **b** The relative autophosphorylation levels of FLAG-SlBRI1, FLAG-T1050A, FLAG-T1050D, and FLAG-K916E proteins in vitro. The autophosphorylation level of FLAG-SlBRI1 was defined as “1”. Data are the means ± SDs of 3 independent measurements. **c** Autophosphorylation level of SlBRI1 in vivo. Autophosphorylation activity of SlBRI1-green fluorescent protein (GFP), T1050A-GFP, T1050D-GFP, and K916E-GFP proteins was detected using anti-pThr antibodies; anti-GFP antibodies were used to show the loading levels for western blotting. **d** The relative autophosphorylation levels of SlBRI1-GFP, T1050A-GFP, T1050D-GFP, and K916E-GFP proteins in vivo. The autophosphorylation level of SlBRI1-GFP was defined as “1”. Data are the means ± SDs of 3 independent measurements

With a function similar to that of a receptor-like kinase, autophosphorylation plays a vital role in BR signal transduction and the biological function of SlBRI1. To further determine whether Thr-1050 phosphorylation influences SlBRI1 autophosphorylation, SlBRI1, T1050A, and T1050D (threonine-1050-aspartic acid) autophosphorylation levels were compared both in vivo and in vitro. The pFLAG-MAC vector containing the cytoplasmic domains of SlBRI1, T1050A, and T1050D was used for the in vitro analysis. The results showed that mutating Thr-1050-D negatively influenced SlBRI1 autophosphorylation, as FLAG-T1050A showed the strongest intensity of phosphorylation band, phosphorylation level of which was 2.5- and 2.2-fold that of FLAG-T1050D and FLAG-SlBRI1, respectively (Fig. 5 a, b).

To further compare autophosphorylation in vivo, proteins from *Nicotiana benthamiana* leaves expressing SlBRI1-GFP, T1050A-GFP, and T1050D-GFP were extracted and examined. As shown in Fig. 5 c and Fig. 5 d, the phosphorylation levels of T1050A-GFP and SlBRI1-GFP were 48 and 23% stronger than that of T1050D-GFP, respectively. Collectively, in vitro and in vivo autophosphorylation assays both suggested a negative role for Thr-1050 phosphorylation in SlBRI1 autophosphorylation.

## Discussion

Many studies have demonstrated the conserved role of BRI1 in plant growth. Loss-of-function *bri1* mutants exhibit general characteristics, such as a dwarf phenotype, dark-green leaves, and infertility [6, 30]. Conversely, overexpression of *BRI1* promotes plant germination, flowering and seed yield and accelerates ripening [24]. Our research aims to examine the effects of SlBRI1 phosphorylation sites on tomato agronomic traits. To explore conservation of Thr-1050 in SlBRI1, the protein sequences of BRI1 homologues from six plant species, SlBRI1 (accession No. NP_001296180.1), StBRI1 (*Solanum tuberosum*, accession No. XP_006357355.1), BRI1 (*Arabidopsis thaliana*, accession No. NP_195650.1), OsBRI1 (*Oryza sativa*, accession No. NP_001044077.1), TaBRI1 (*Triticum aestivum*, accession No. DQ_655711.1), and ZmBRI1 (*Zea mays*, accession No. XP_008656807.1), were selected for comparison. Multiple sequence alignment revealed Thr-1050 to be highly conserved among these plant species, suggesting important roles in BR signalling and plant growth (Additional file 4: Figure S4). In Arabidopsis, Thr-1045 is equivalent to Thr-1050 in the tomato protein. Previous studies have suggested that BRI1 Thr-1045 is likely to positively regulate BR signalling and plant growth, as unphosphorylated Ser-1044 or Thr-1045 result in a dwarf phenotype and reduced BR signal transduction [31]. We generated transgenic tomato lines expressing SlBRI1 or T1050A, and the results revealed that Thr1050 acts as a negative regulator of growth in this crop. Compare with *cu3*^*-abs1*^ and *cu3*^*-abs1*^ plants harbouring SlBRI1, plants in which Thr-1050 was dephosphorylated exhibited greater plant expansion, larger leaf area, earlier flowering and ripening and higher yields (Figs. 1, 2). These findings indicate that Thr-1050 functions in opposite manners in tomato and Arabidopsis.

Plant yield is one of the most important agronomic traits in tomato production, and BRs are considered growth promoters that can increase agricultural yields. Our results showed that the yield of individual P_*SlBRI1*_::T1050A-GFP plants was 1.8-fold that of P_*SlBRI1*_::SlBRI1-GFP plants and 3.4-fold that of *cu3*^*-abs1*^ plants. This increased yield resulted from greater flower numbers per cluster and greater fruit weight (Fig. 2). However, as the physiological limitation of fruit yield is restricted by the photosynthetic capacity of the plant, a greater number of flowers is usually not necessary to achieve higher yields [32]. Indeed, a similar result has been reported in which overexpression of *SlBRI1* promoted individual plant yield by increasing flower numbers, even though fruit weight was reduced [26]. To investigate this phenomenon, we evaluated characteristics related to photosynthesis. P_*SlBRI1*_::T1050A-GFP plants exhibited similar plant heights and CO_2_ assimilation rates but larger leaf areas and greater plant expansion. As the degree of increase in leaf area was greater than that of plant expansion, P_*SlBRI1*_::T1050A-GFP plants had a larger leaf area index and produced more photosynthate for blossoming and fruiting. Furthermore, research in Arabidopsis has shown that by regulating the number of branches and effective siliques, BRI1 residues Ser-1042, Ser-1168, Ser-1172, Ser-1179, and Thr-1180 can influence individual plant yield [33]. Therefore, we conclude that the regulatory role of Thr-1050 in individual plant yield differs from that of SlBRI1, as Thr-1050 controls both inflorescence architecture and also fruit expansion via the leaf area index.

Crop yield depends not only on individual plant yield but also on plant density. Previous studies have reported that partially suppressing expression of *OsBRI1* increases yields by 30% because of the relatively more compact plant architecture and higher planting density [19]. Similarly, Ser-1042 in BRI1 also affects seed yield, as S1042A mutants present normal seed yield but relatively more compact plant architecture [33]. In our research, expansion in P_*SlBRI1*_::T1050A-GFP was 1.2-fold that in P_*SlBRI1*_::SlBRI1-GFP; thus, the planting density of P_*SlBRI1*_::T1050A-GFP was 83% of that of P_*SlBRI1*_::SlBRI1-GFP. However, the individual yield of P_*SlBRI1*_::T1050A-GFP plants was 1.8-fold that of P_*SlBRI1*_::SlBRI1-GFP plants (Figs. 1, 2). These two parameters functioned together to increase the yield of P_*SlBRI1*_::T1050A-GFP to approximately 150% of that of P_*SlBRI1*_::SlBRI1-GFP per area, suggesting high yield potential for Thr-1050 in crop breeding.

Tomato fruit quality can also be regulated by BR signals. Previous studies have revealed that overexpressing *SlBRI1* accelerates fruit ripening and increases the contents of carotenoids, soluble solids, AsA, and soluble sugars by promoting ethylene production [26]. Moreover, the transcription factor BZR1, which is involved in the BR response, was found to regulate fruit quality, whereby heterologous expression of *BZR1* in tomato resulted in increased levels of carotenoids, soluble solids, AsA and soluble sugars in fruits [34]. In our research, P_*SlBRI1*_::T1050A-GFP fruits ripened earlier when compared with P_*SlBRI1*_::SlBRI1-GFP fruits and presented similar levels of ethylene production, soluble solid contents and carotenoid contents (data not shown) but lower contents of AsA, soluble sugars, and malic acid and less firmness. In contrast, P_*SlBRI1*_::T1050A-GFP fruits presented greater levels of citric acid and a higher shape index (Fig. 3). Therefore, we can conclude that the regulatory role of Thr-1050 in fruit quality differs from that of SlBRI1. The promotion of *SlBRI1* during tomato ripening was due mostly to increased ethylene evolution; however, because the endogenous ethylene content was unchanged, lack of phosphorylation at Thr1050 affected fruit quality independently of ethylene. These functional differences between Thr-1050 and SlBRI1 in terms of fruit quality may result from their own differences. Moreover, we used the *SlBRI1* native promoter in our research, whereas the constitutive cauliflower mosaic virus 35S promoter was used in previous research, which also may have contributed to these differences.

Previous studies have demonstrated that BR signals play regulatory roles in BR biosynthesis and BR sensitivity and that BR mutants in BR signal show BR insensitivity and higher levels of BR biosynthesis gene expression [27, 35–38]. As a BR receptor, SlBRI1 affects plant growth by regulating BR signal transduction in tomato; transgenic plants overexpressing *SlBRI1* exhibited increased BR signalling and consequently had longer hypocotyls and reduced transcription of BR biosynthesis genes *SlCPD* and *SlDWARF* [26]. Consistent with previous findings, the P_*SlBRI1*_::T1050A-GFP plants in our study showed stronger BR signal intensity on the basis of the following evidence. First, the expression levels of *SlCPD* and *SlDWARF*, which should be feedback-inhibited by BR signals [37, 38], were similar between P_*SlBRI1*_::T1050A-GFP and P_*SlBRI1*_::SlBRI1-GFP plants and lower than those of *cu3*^*-abs1*^. Second, the BR contents of P_*SlBRI1*_::T1050A-GFP plants were equal to those of P_*SlBRI1*_::SlBRI1-GFP plants and lower than those of *cu3*^*-abs1*^. Third, the hypocotyls of P_*SlBRI1*_::T1050A-GFP were longer than those of P_*SlBRI1*_::SlBRI1-GFP under dark culture conditions. Finally, P_*SlBRI1*_::T1050A-GFP plants were more sensitive to BR because their relative hypocotyl lengths were longer than those of P_*SlBRI1*_::SlBRI1-GFP under dark culture conditions with epi-BL treatment. Thus, we can conclude that the recovery capability of T1050A was stronger than that of SlBRI1 with respect to BR signal intensity, which may partly explain the different phenotypes between P_*SlBRI1*_::T1050A-GFP and P_*SlBRI1*_::SlBRI1-GFP (Fig. 4).

BRI1 has been shown to function as a serine/threonine/tyrosine protein kinase that transduces BR signals via phosphorylation sites [5]. Previous studies in Arabidopsis have shown that Thr-1045 of BRI1, which is analogous to Thr-1050 of SlBRI1, might be essential for BRI1 kinase function because the mutation either S1044A or T1045A nearly abolished BR signalling and exhibited a dwarf phenotype with respect to autophosphorylation levels in vitro [31]. In contrast, our results showed a higher level of T1050A autophosphorylation compared to SlBRI1 both in vivo and in vitro (Fig. 5). A previous study also reported that compared with SlBRI1, T1050A retained full kinase activity in vitro [29]. Thus, we demonstrate that Thr-1050 might regulate plant growth by actively affecting the kinase function of SlBRI1, and this pattern is opposite to that of BRI1 Thr-1045 in Arabidopsis.

In addition, structural changes in the SlBRI1 protein resulting from the substitution of Thr-1050 to Ala-1050 may also affect kinase function. To investigate this phenomenon, three-dimensional (3D) protein folding structure prediction and subcellular localization of T1050A and SlBRI1 were explored. The results showed that dephosphorylation of Thr-1050 did not alter the protein structure or subcellular localization of SlBRI1, which was consistent with the localization of SlBRI1 to the plasma membrane (Additional files 5, 6: Figures. S5, S6). Additional studies to identify the related interacting proteins and downstream target genes are still needed to understand the regulatory mechanisms of Thr-1050.

## Conclusions

BRs compose a group of phytohormones that regulate various biological processes at low concentrations. However, it is difficult to precisely control BR signal strength, and attempts at BR signal manipulation can cause unstable effects and result in the failure to reach full BR potential. *BRI1* correlated with the levels of both expression and phosphorylation of components involved in BR signal transduction, and the functions of the specific serine/threonine/tyrosine residues within BRI1 vary with Arabidopsis growth. However, the associated agronomic traits and molecular mechanisms of SlBRI1 phosphorylation sites remain unclear in tomato. Our results reveal the biological function of Thr-1050 in tomato. Transgenic tomato plants harbouring T1050A exhibited stronger BR signal strength, higher autophosphorylation levels, more vigorous vegetative development, higher yields, and lower fruit nutrient contents. These results highlight the potential of SlBRI1 phosphorylation sites in tomato breeding and provide a molecular basis for establishing high-yielding tomato varieties via the precise control of BR signalling.

## Methods

### Site-directed mutagenesis and vector construction

To construct the plant expression vector expressing wild-type *SlBRI1* (P_*SlBRI1*_::SlBRI1-GFP-pBI121), the full-length *SlBRI1* gene (Solyc04g051510) without its stop codon and its promoter from tomato (*Solanum lycopersicum* cv. Moneymaker) were amplified. The native promoter of *SlBRI1* (2989 bp) was first cloned into the *Hind* III and *Xba* I sites of the binary pBI121 vector (CLONTECH, Palo Alto, CA) to replace the 35S promoter. The amplified *SlBRI1* was then recombined into pBI121 with a GFP-encoding sequence followed by its CT region. To obtain plant expression vectors expressing the T1050A (in which Thr-1050 was replaced with alanine) or T1050D (in which Thr-1050 was replaced with aspartic acid) mutation of SlBRI1, P_*SlBRI1*_::SlBRI1-GFP-pBI121 was used as a template, and site mutations of T1050A and T1050D were generated by overlapping PCR amplification. After sequencing verification, all constructs were transformed into *Agrobacterium tumefaciens* strain GV3101 for tomato transformation.

The full-length wild-type SlBRI1, T1050A, and T1050D sequences were amplified from plant expression vectors and cloned into the CaM35S-GFP vector for phosphorylation analysis in vivo [39]. The cytoplasmic domains (815 aa to 1196 aa) of SlBRI1, T1050A, and T1050D were also amplified from plant expression vectors and cloned into the pFLAG-MAC vector (Sigma-Aldrich Saint Louis, MO, USA) for phosphorylation analysis in vitro. A kinase-inactive mutant generated by a lysine-916-glutamic acid (K916E) substitution of the invariant residue was designed as described above and used as a negative control in the phosphorylation analysis [29]; the conserved Lys residues of this mutant lacks kinase activity both in vitro and in vivo. All of the primers used in this study are listed in Supplementary Table S1 (Additional file 7).

### Tomato transformation

With respect to tomato transformation, *cu3*^*-abs1*^ was used as a transgenic acceptor in accordance with the cotyledon transformation method. Three independent homozygous P_*SlBRI1*_::T1050A lines (P_*SlBRI1*_::T1050A-1, P_*SlBRI1*_::T1050A-7, and P_*SlBRI1*_::T1050A-10) and one dependent homozygous P_*SlBRI1*_::SlBRI1–1 line were used in this study. All transgenic lines were advanced to the T_2_ generation.

### Response of hypocotyl elongation to exogenous BL

Seeds of the *cu3*^*-abs1*^ and transgenic lines were sterilized and then inoculated in culture bottles containing 1/2 MS medium (1/2 strength Murashige and Skoog medium containing 0.75% agar and 2% sucrose, pH 5.8) containing 0 nM, 5 nM, 50 nM, or 500 nM epi-BL; the bottles were placed in the dark for 9 days at 25 °C. Hypocotyl lengths were measured to the nearest 0.1 cm; the relative hypocotyl length was the relative change of hypocotyl length at different concentrations of epi-BL. A minimum of 15 seedlings from each treatment were randomly examined.

### Agronomic trait characterization

For agronomic trait investigation, all tomato plants, including those of transgenic lines and *cu3*^*-abs1*^ lines, were grown in a glasshouse under natural daylight and temperature conditions. Agronomic traits involved in vegetative development were measured at the fruit-ripening stage (120 days after sowing) in accordance with the following rules: plant height was considered the distance from the ground to the top of the plant; stem diameter and internodal distance were considered the diameter and length of the second flower node, respectively; plant expansion was considered the maximum diameter of the plant; leaf area and stem-leaf angle were measured on the sixth leaf; leaf area was measured by scanning followed by calculations using Image J software; and the stem-leaf angle referred to the angle between the leaf and stem. At least 5 plants per transgenic and *cu3*^*-abs1*^ line were used for each characteristic.

Agronomic traits involved in reproductive development were measured in accordance with the following rules: fruit weight was considered the individual fruit weight at the RR stage; the fruit number per cluster was considered the number of the third cluster; the fruit shape index was the ratio of the longitudinal diameter to the transverse diameter at the fruit breaker stage; the yield per plant was considered the total weight of the first to the fourth fruit nodes; the pericarp thickness at the fruit yellow ripening stage was measured using Vernier callipers; fruit firmness was measured using a hand-held penetrometer (FHM-1, Japan); and the seed number was considered the number of seeds per fruit. At least 15 independent biological replicates were used for each characteristic.

### Scanning Electron microscope observation

At least 9 sections of the sixth leaf from both transgenic and *cu3*^*-abs1*^ plants were isolated to observe the cell size and cell number via scanning electron microscopy. Sample preparation was performed as previously described, and the samples were photomicrographed using an S-4800 scanning electron microscope (Hitachi, Japan) [40]. Cell size was compared under a 1500x-magnified visual field with the scanning electron microscope, and the numbers of cells per unit leaf area were counted under a 300x-magnified visual field.

### Quantitative real-time PCR analysis

Total RNA from various tissues of transgenic and *cu3*^*-abs1*^ plants was extracted using an RNAiso Plus kit (TaKaRa, Dalian, China) and transcribed to cDNA with Transcriptor First Strand cDNA Synthesis Kit (Roche, Mannheim, Germany) according to the manufacturer’s protocol. qRT-PCR was performed using a SYBR Green Master Mix kit (Vazyme, Nanjing, China), as previously described [41]. The tomato *UBI3* gene was used as an internal control, and all primers are listed in Table S1 [26, 42] (Additional file 7). The amplification efficiency of each primer combination was checked by standard-curve analysis. Each sample was represented by 3 biological replicates and technical replications.

### Western blot analysis

Total proteins were extracted from 25-day-old tomato seedlings (0.2 g) expressing wild-type SlBRI1, Thr1050A, T1050D, or K916E and mixed with 2x SDS gel loading buffer. The procedure was performed as previously described [31].

### Analysis of CO_2_ assimilation rates

The sixth leaf from both transgenic and *cu3*^*-abs1*^ plants was selected to measure the CO_2_ assimilation rate (Pn) using an infrared gas analyser-based portable photosynthesis system (LI-6800; LI-COR, Lincoln, NE, USA). The CO_2_ concentration and PPFD used for measurement were 400 μmol mol^− 1^ and 800 μmol m^− 2^ s^− 1^, respectively.

### Determination of BR contents

The third leaf (0.5 g) from both transgenic and *cu3*^*-abs1*^ plants was harvested to measure BR using a BR ELISA Kit (MyBiosource, San Diego, USA, Cat. #MBS9364120) according to the manufacturer’s instructions. The results for each line were quantified with 3 independent biological samples.

### Determination of ascorbic acid, soluble sugars, organic acids, and soluble solid contents

Fruits at the YR and RR stages were harvested for analysis. AsA extraction and analyses were performed as previously described [43]. Soluble sugars were extracted from 0.5 g of fruit pericarp tissue, mixed with 15 ml of distilled water and subsequently heated in a boiling water bath for 20 min. The solution was cooled to room temperature and centrifuged at 4000×g for 10 min. Afterward, the supernatant was diluted to 25 ml and then subjected to HPLC analysis (1100 RID, Agilent, Palo Alto, CA, USA) using an Agilent Zorbax chromatogram column (4.6 × 150 mm, 5 μm, Agilent, Palo Alto, CA, USA, PN. 843,300–908). Organic acid extractions were performed as previously described [44], and detection was performed by HPLC (1260 RID, Agilent, Palo Alto, CA, USA) using an Agilent Zorbax SB-C18 chromatogram column (4.6 × 150 mm, 5 μm, Agilent, Palo Alto, CA, USA, PN. 883,975–902). The soluble solid content of fresh fruit juice was detected using a hand-held refractometer (Chengdu Optical Instrument Factory, Chengdu, China). The results for each line were quantified via external calibration, with 3 independent biological samples and 3 technical replications.

### Autophosphorylation analysis

For autophosphorylation analyses in vitro, constructs expressing the cytoplasmic domain of wild-type SlBRI1, T1050A, T1050D, and K916E were subcloned into a pFLAG-MAC vector, which were then transformed into *E. coli* BL21 (DE3) pLysS (Transgene, Beijing, China). The methods of protein purification and autophosphorylation assays as previously described [31, 45]. Autophosphorylation activity of recombinant proteins was detected using anti-pThr antibodies (CST, Danvers, MA, USA, Cat. #93815), and aliquots of the recombinant proteins were detected using anti-FLAG antibodies (Transgene, Beijing, China). The experiments were repeated 3 times and the results were consistent. Intensities of unsaturated bands were quantified using ImageJ software and presented as relative values compared with the FLAG-SlBRI1.

For autophosphorylation analyses in vivo, strain GV3101 containing the indicated construct (wild-type SlBRI1-GFP, T1050A-GFP, T1050D-GFP, or K916E-GFP) was grown overnight in liquid LB medium supplemented with the appropriate antibiotics. The cultures were centrifuged, and the cells were resuspended in 10 mM MgCl_2_, 10 mM MES-KOH, and 200 μM AS to a final OD_600_ = 0.3–0.5. The indicated cultures were infiltrated into 3-week-old *N. benthamiana* leaves using a syringe. *N. benthamiana* leaves without the main vein were subsequently harvested at 48 h after infection. The leaves were ground to fine powder in liquid nitrogen, after which 5 ml of 2x extraction buffer [50 mM Tris-HCl, pH 7.4; 150 mM NaCl; 10% glycerol; 5 mM EDTA, pH 8.0; 20 mM NaF; 1 mM PMSF; 0.2% (v/v) Triton X-100; 1% (w/v) PVPP; and 2% (m/v) protease inhibitor cocktail (Roche)] was added. The samples were clarified by centrifugation at 13000 g for 15 min at 4 °C, after which the protein concentration was adjusted to 2 mg ml^− 1^ with 0.8x extraction buffer. Immunoprecipitation was performed using 1.5 ml of total protein by adding 10 μl of anti-GFP (1 μg μl^− 1^) (Transgene, Beijing, China) followed by incubation at 4 °C for 3–4 h. Afterward, 40 μl of protein G magnetic beads (NEB) was added, and the sample was incubated at 4 °C for 1–2 h. The beads were subsequently washed 4 times with TBS, immunoprecipitated, eluted with 40 μl of 2x SDS loading buffer and boiled for 5 min. Anti-GFP was used to show the loading levels, while anti-pThr was used to determine the autophosphorylation level in vivo. The experiments were repeated 3 times and the results were consistent. Intensities of unsaturated bands were quantified using ImageJ software and presented as relative values compared with the SlBRI1-GFP.

### Statistical analysis

The data in this study were analysed with SPSS version 17.0 and Student’s t-tests. Mean and standard error values were calculated, and *P* < 0.05 or 0.01 was considered statistically significant in comparisons with P_*SlBRI1*_::SlBRI1-GFP plants.

## Additional files

Additional file 1: **Figure S1.** Expression of transgenic SlBRI1 proteins and phenotypes of P_*SlBRI1*_::SlBRI1-GFP transgenic lines. Top, phenotypes of plants at the maturation stage (120 days after sowing). Bottom, western blot analysis of transgenic SlBRI1 expression using anti-green fluorescent protein (GFP) antibodies. CBB, Coomassie brilliant blue. (JPG 604 kb)

Additional file 2: **Figure S2.** Thr-1050 did not alter photosynthesis. The leaf CO_2_ assimilation rate (Pn) of the sixth leaf at the maturation stage. Data are the means ± SDs of 3 independent biological samples. (PDF 1080 kb)

Additional file 3: **Figure S3.** Total soluble solids in fruits. (PDF 188 kb)

Additional file 4: **Figure S4.** Alignment of the partial kinase domain sequences of SlBRI1, StBRI1, AtBRI1, OsBRI1, TaBRI1 and ZmBRI1. SlBRI1 (*Solanum lycopersicum*, accession No. NP_001296180.1), StBRI1 (*Solanum tuberosum*, accession No. XP_006357355.1), BRI1 (*Arabidopsis thaliana*, accession No. NP_195650.1), OsBRI1 (*Oryza sativa*, accession No. NP_001044077.1), TaBRI1 (*Triticum aestivum*, accession No. DQ_655711.1), ZmBRI1 (*Zea mays*, accession No. XP_008656807.1). (PDF 1162 kb)

Additional file 5 **Figure S5.** Three-dimensional (3D) folding structure prediction of SlBRI1 and T1050A. The 3D folding structure was predicted using SWISS-MODEL. (PDF 1687 kb)

Additional file 6: **Figure S6.** Subcellular localization of SlBRI1 and T1050A. *Agrobacterium*-mediated transient transformation of tobacco epidermal cells. First line, subcellular localization of GFP. Second line, subcellular localization of SlBRI1-GFP. Third line, subcellular localization T1050A-GFP. Left panels: bright-field images. Middle panels: green fluorescence signal under blue light. Right panels: merged images. Scale bars, 50 μm. (JPG 932 kb)

Additional file 7: **Table S1.** Primers used in this research. (DOCX 21 kb)

## Data Availability

All data generated or analysed during this study are included in this published article and its additional files. All plant materials were obtained from Northwest A&F University, Yangling, Shaanxi, China.

## Abbreviations

35S: Constitutive cauliflower mosaic virus 35S promoter
AsA: Ascorbic acid
BAK1: BRI1-ASSOCIATED RECEPTOR KINASE1
BES1: BRI1-EMS SUPPRESSOR1
BR: Brassinosteroid
BRI1: BRASSINOSTEROID INSENSITIVE1
BZR1: BRASSINAZOLERESISTANT1
CBB: Coomassie brilliant blue
CPD: CONSTITUTIVE PHOTOMORPHOGENESIS AND DWARF
CT: C-terminal domain
DWARF: 6-DEOXOCASTASTERONE OXIDASE
ELISA: Enzyme-linked immunosorbent assay
epi-BL: 24-epibrassinolide
FW: Fresh weight
GFP: Green fluorescent protein
HPLC: High-performance liquid chromatography
JM: Juxtamembrane region
K916E: Lysine-916-Glutamic acid
KD: Kinase domain
MS: Murashige and Skoog medium
PMSF: Phenylmethane sulfonyl fluoride
Pn: Net photosynthetic rate
PVPP: Crosslinking polyvinylpyrrolidone
qRT-PCR: Quantitative real-time PCR
RR: Red ripening
SDS-PAGE: Sodium dodecyl sulfate-polyacrylamide gel electrophoresis
Ser: Serine
T1050A: Threonine-1050-alanine
T1050D: Threonine-1050-aspartic acid
TBS: Tris-buffered saline
Thr: Threonine
Tris: Tris (hydroxymethyl) aminomethane
Tyr: Tyrosine
UBI3: UBIQUITIN/RIBOSOMAL FUSION PROTEIN 3
YR: Yellow ripening
