# Supplementary figures and images for: Modification of Threonine-1050 of SlBRI1 regulates BR Signalling and increases fruit yield of tomato
Source: BMC Plant Biol. 2019 Jun 13;19:256. doi: 10.1186/s12870-019-1869-9 (PMC6567510; doi:10.1186/s12870-019-1869-9)

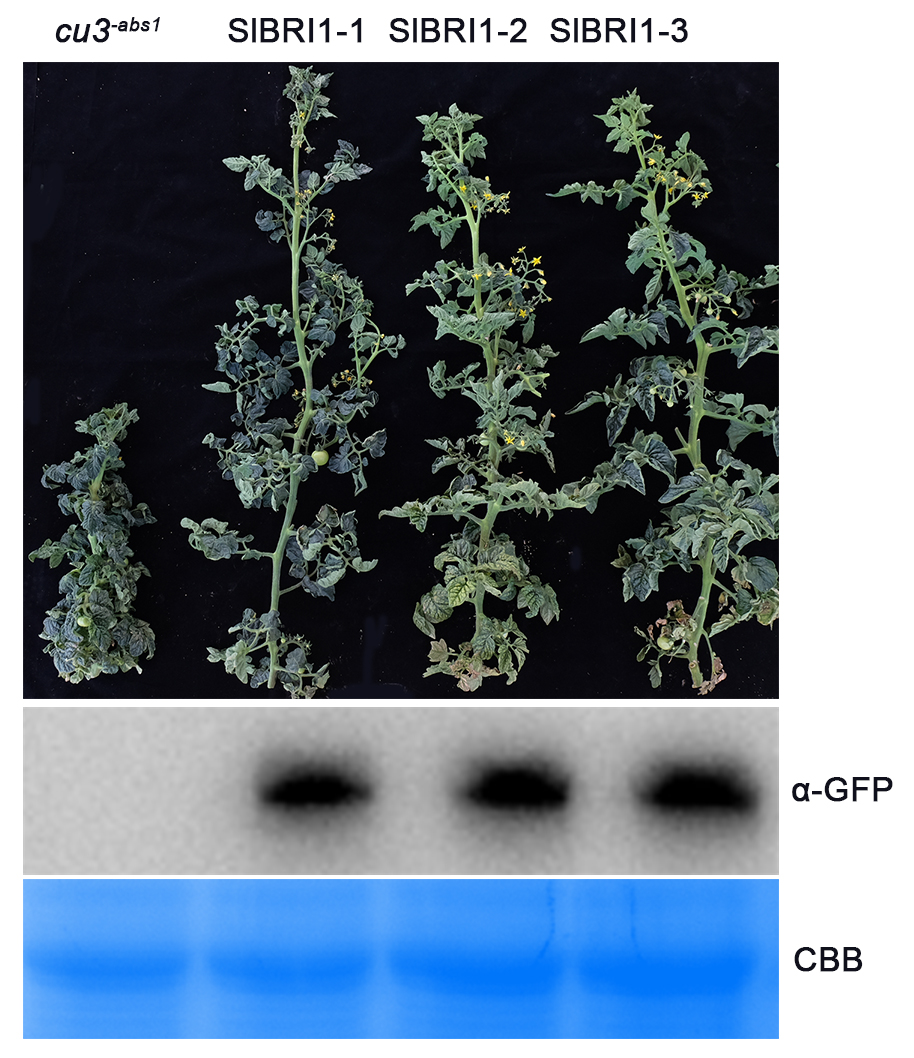

Supplement: Supplementary file 1 — Figure S1. Expression of transgenic SlBRI1 proteins and phenotypes of PSlBRI1::SlBRI1-GFP transgenic lines. Top, phenotypes of plants at the maturation stage (120 days after sowing). Bottom, western blot analysis of transgenic SlBRI1 expression using anti-green fluorescent protein (GFP) antibodies. CBB, Coomassie brilliant blue. (JPG 604 kb) [file 12870_2019_1869_MOESM1_ESM.jpg]

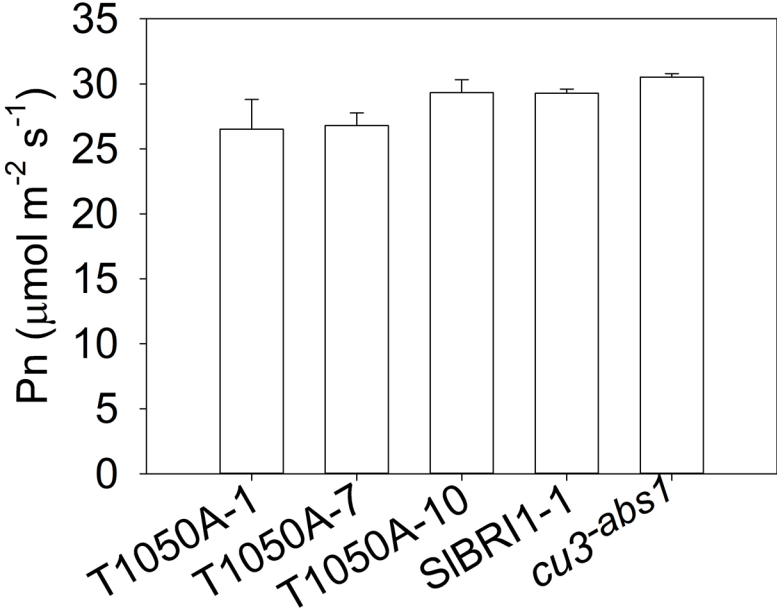

Supplement: Supplementary file 2 — Figure S2. Thr-1050 did not alter photosynthesis. The leaf CO2 assimilation rate (Pn) of the sixth leaf at the maturation stage. Data are the means ± SDs of 3 independent biological samples. (PDF 1080 kb) [file 12870_2019_1869_MOESM2_ESM.pdf]

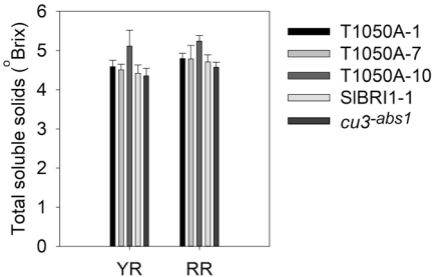

Supplement: Supplementary file 3 — Figure S3. Total soluble solids in fruits. (PDF 188 kb) [file 12870_2019_1869_MOESM3_ESM.pdf]

SIBRI1

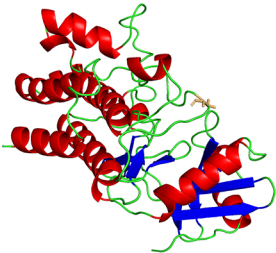

T1050A

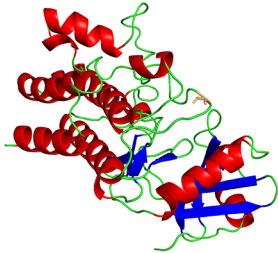

Supplement: Supplementary file 5 — Figure S5. Three-dimensional (3D) folding structure prediction of SlBRI1 and T1050A. The 3D folding structure was predicted using SWISS-MODEL. (PDF 1687 kb) [file 12870_2019_1869_MOESM5_ESM.pdf]

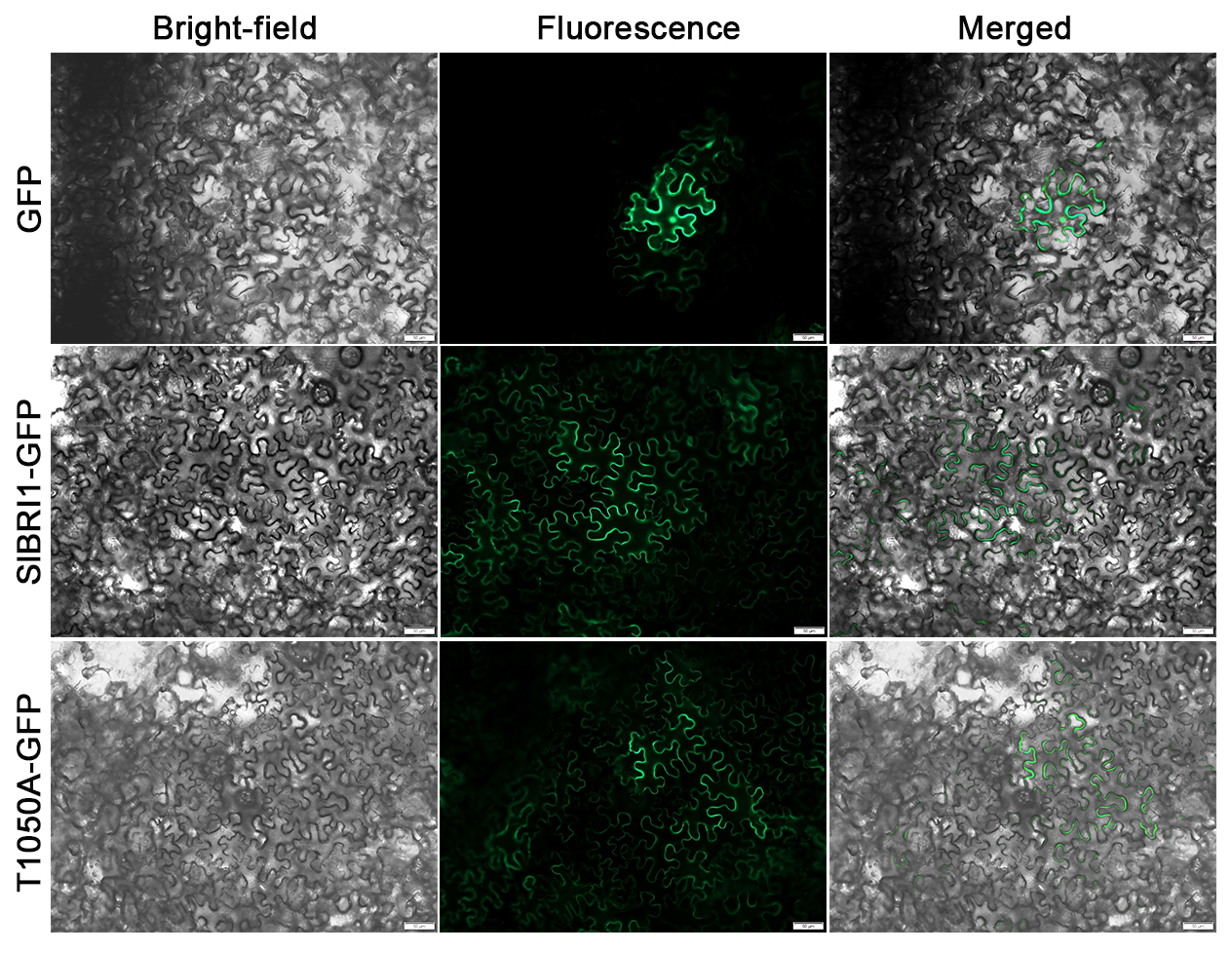

Supplement: Supplementary file 6 — Figure S6. Subcellular localization of SlBRI1 and T1050A. Agrobacterium-mediated transient transformation of tobacco epidermal cells. First line, subcellular localization of GFP. Second line, subcellular localization of SlBRI1-GFP. Third line, subcellular localization T1050A-GFP. Left panels: bright-field images. Middle panels: green fluorescence signal under blue light. Right panels: merged images. Scale bars, 50 μm. (JPG 932 kb) [file 12870_2019_1869_MOESM6_ESM.jpg]
