# Supplementary material for: Modification of Threonine-1050 of SlBRI1 regulates BR Signalling and increases fruit yield of tomato
Source: BMC Plant Biol. 2019 Jun 13;19:256. doi: 10.1186/s12870-019-1869-9 (PMC6567510; doi:10.1186/s12870-019-1869-9)
Supplement: Supplementary file 4 — Figure S4. Alignment of the partial kinase domain sequences of SlBRI1, StBRI1, AtBRI1, OsBRI1, TaBRI1 and ZmBRI1. SlBRI1 (Solanum lycopersicum, accession No. NP_001296180.1), StBRI1 (Solanum tuberosum, accession No. XP_006357355.1), BRI1 (Arabidopsis thaliana, accession No. NP_195650.1), OsBRI1 (Oryza sativa, accession No. NP_001044077.1), TaBRI1 (Triticum aestivum, accession No. DQ_655711.1), ZmBRI1 (Zea mays, accession No. XP_008656807.1). (PDF 1162 kb) [file 12870_2019_1869_MOESM4_ESM.pdf]

```
SlBRI1   1038-LMSAMDTHLSVSTLAGTPGYVPPEYYQSFRCSTKGDVYSYGVVLLELLTG-1087
StBRI1   1037-LMSAMDTHLSVSTLAGTPGYVPPEYYQSFRCSTKGDVYSYGVVLLELLTG-1086
AtBRI1   1033-LMSAMDTHLSVSTLAGTPGYVPPEYYQSFRCSTKGDVYSYGVVLLELLTG-1082
OsBRI1    957-LMSVVDTHLSVSTLAGTPGYVPPEYYQSFRCTTKGDVYSYGVVLLELLTG-1006
TaBRI1    960-MMSVVDTHLSVSTLAGTPGYVPPEYYQSFRCTTKGDVYSYGVVLLEPLTG-1009
ZmBRI1    958-MMSVVDTHLSVSTLAGTPGYVPPEYYQSFRCTTKGDVYSYGVVLLELLTG-1007
```
