# Supplementary material for: Modification of Threonine-1050 of SlBRI1 regulates BR Signalling and increases fruit yield of tomato
Source: BMC Plant Biol. 2019 Jun 13;19:256. doi: 10.1186/s12870-019-1869-9 (PMC6567510; doi:10.1186/s12870-019-1869-9)
Supplement: Supplementary file 7 — Table S1. Primers used in this research. (DOCX 21 kb) [file 12870_2019_1869_MOESM7_ESM.docx]

Table S1. Primers used in this research

| Gene | Forward primer (5’-3’) | Reverse primer (5’-3’) | Reference |
| --- | --- | --- | --- |
| Promoter of *SlBRI1*  *SlBRI1* for tomato transformation  T1050A for site mutation  T1050D for site mutation  K916E for site mutation  *SlBRI1* and *slbri1* for phosphorylation analysis in vitro  *SlBRI1* and *slbri1* for phosphorylation analysis in vivo  *SlCPD* for qRT-PCR | gaccatgattacgccAAGCTTCTCCATTTCAATTATTGCTCAAAGG  tctttgaagtctagagCTCGAGATGAAAGCTCACAAAACTGTGTTTAAC  TGAGTGTCAGCgCTCTTGCCGG  TGAGTGTCAGCGATCTTGCCGG  GAGTGTTGTAGCTATTGAGAAATTGATACACG  GACGATGACAAAGTCAAGCTTgagacgaagaagaggaggagg  GAGCTCGGTACCCGGGGATCCATGAAAGCTCACAAAACTGTGTTTAAC  CTTCTCTCCGAGCTGTTCATCTAG | ACCACCCGGGGATCCTCTAGACTTCAAAGATTGAAACTTTATAGCTTAAA  GCCCTTGCTCACCATGGTACCCTTATCGTCGTCATCCTTGTAATC  CCGGCAAGAGCGCTGACACTCA  CCGGCAAGATCGCTGACACTCA  CGTGTATCAATTTCTCAATAGCTACAACACTC  TCTGCAGGTACCCGGGAATTCAAGGTGTTTGCTCAGCTCATTG  CTTGCTCACCATGGTGTCGACAAGGTGTTTGCTCAGCTCATTG  GAAGGAAAACAGAGAGTTCCACTC | This Study  This Study  This Study  This Study  This Study  This Study  This Study  (Peng *et al*., 2014) |
| *SlDWARF* for qRT-PCR | AAAATTGATGAGTTTATGAGATCCC | CAAGCATATCATGTTGAATTTCCT | (Nie *et al*., 2017) |
| *SlBRI1* for qRT-PCR | TTCAATGGCACGATCCCGAA | TGGGGAGAGGATACCCACAG | (Nie *et al*., 2017) |
| *UBI3* for qRT-PCR | GTGTGGGCTCACCTACGTTT | ACAATCCCAAGGGTTGTCAC | (Nie *et al*., 2017) |
